# Role of cardiac magnetic resonance in stratifying arrhythmogenic risk in mitral valve prolapse patients: a systematic review and meta-analysis

**DOI:** 10.1007/s00330-024-10815-3

**Published:** 2024-06-06

**Authors:** Marco Gatti, Ambra Santonocito, Francesco Pio Papa, Fabrizio D’Ascenzo, Ovidio De Filippo, Guglielmo Gallone, Anna Palmisano, Lorenzo Pistelli, Gaetano Maria De Ferrari, Antonio Esposito, Carla Giustetto, Paolo Fonio, Riccardo Faletti

**Affiliations:** 1 Department of Surgical Sciences, Radiology Unit, University of Turin, Turin, Italy; 2 Department of Biomedical Imaging and Image-Guided Therapy, Medical University of Vienna, Vienna, Austria; 3 Division of Cardiology, Department Cardiovascular and Thoracic, Città della Salute e della Scienza Hospital, University of Turin, Turin, Italy; 4 Clinical and Experimental Radiology Unit, Experimental Imaging Center, IRCCS San Raffaele Scientific Institute, Milan, Italy; 5 School of Medicine, Vita-Salute San Raffaele University, Milan, Italy

**Keywords:** Mitral valve prolapse, Ventricular arrhythmias, Risk stratification, Cardiac magnetic resonance imaging, Fibrosis

## Abstract

**Objectives:**

To perform a systematic review and meta-analysis of studies investigating the diagnostic value of cardiac magnetic resonance (CMR) features for arrhythmic risk stratification in mitral valve prolapse (MVP) patients.

**Materials and methods:**

EMBASE, PubMed/MEDLINE, and CENTRAL were searched for studies reporting MVP patients who underwent CMR with assessment of: left ventricular (LV) size and function, mitral regurgitation (MR), prolapse distance, mitral annular disjunction (MAD), curling, late gadolinium enhancement (LGE), and T1 mapping, and reported the association with arrhythmia. The primary endpoint was complex ventricular arrhythmias (co-VAs) as defined by any non-sustained ventricular tachycardia, sustained ventricular tachycardia, ventricular fibrillation, or aborted sudden cardiac death. Meta-analysis was performed when at least three studies investigated a CMR feature. PROSPERO registration number: CRD42023374185.

**Results:**

The meta-analysis included 11 studies with 1278 patients. MR severity, leaflet length/thickness, curling, MAD distance, and mapping techniques were not meta-analyzed as reported in < 3 studies. LV end-diastolic volume index, LV ejection fraction, and prolapse distance showed small non-significant effect sizes. LGE showed a strong and significant association with co-VA with a LogORs of 2.12 (95% confidence interval (CI): [1.00, 3.23]), for MAD the log odds-ratio was 0.95 (95% CI: [0.30, 1.60]). The predictive accuracy of LGE was substantial, with a hierarchical summary ROC AUC of 0.83 (95% CI: [0.69, 0.91]) and sensitivity and specificity rates of 0.70 (95% CI: [0.41, 0.89]) and 0.80 (95% CI: [0.67, 0.89]), respectively.

**Conclusions:**

Our study highlights the role of LGE as the key CMR feature for arrhythmia risk stratification in MVP patients. MAD might complement arrhythmic risk stratification.

**Clinical relevance statement:**

LGE is a key factor for arrhythmogenic risk in MVP patients, with additional contribution from MAD. Combining MRI findings with clinical characteristics is critical for evaluating and accurately stratifying arrhythmogenic risk in MVP patients.

**Key Points:**

- *MVP affects 2–3% of the population, with some facing increased risk for arrhythmia*.
- *LGE can assess arrhythmia risk, and MAD may further stratify patients*.
- *CMR is critical for MVP arrhythmia risk stratification, making it essential in a comprehensive evaluation*.

## Introduction

Mitral valve prolapse (MVP) is a valvular anomaly characterized by superior displacement of one or both mitral valve leaflets into the left atrium [1] which affects approximately 2–3% of the general population [2].

While, in unselected cohorts, the prognosis is mainly dictated by the severity of mitral regurgitation (MR), a subgroup of patients is at risk of malignant ventricular arrhythmias (VA) and sudden cardiac death (SCD). The incidence of SCD in the community MVP population is low and between 0.1% and 0.4% per year [3, 4]

However, a subgroup of patients in retrospect defined as affected by “Arrhythmic mitral valve prolapse” (AMVP) [5] may be at substantially higher SCD risk. The characteristics of AMVP are poorly characterized, accordingly, the diagnostic strategies to identify these patients represent an unmet need. A comprehensive non-invasive arrhythmic risk stratification of patients with MVP might allow for tailor monitoring (i.e., implantable loop recorder) and preventive strategies (beta-blockers and implantable cardioverter defibrillator) in high-risk patients [5].

Clinical, Electrocardiogram (ECG), and imaging characteristics including syncope, T-wave inversion, longer QTc interval, ventricular arrhythmia burden and complexity, bi-leaflet prolapse, longer anterior mitral valve leaflet, mitral annular disjunction (MAD), and late gadolinium enhancement (LGE) have previously been associated with complex ventricular arrhythmias (co-VAs) in MVP patients [6].

According to the 2021 ESC/EACTS Guidelines for the management of valvular heart disease (VHD) [7], significant gaps in evidence exist in the following elements of VHD concerning the relationship between MR, SCD, and VA. In addition, it has been emphasized that non-invasive assessment with three-dimensional echocardiography, cardiac computed tomography, cardiac magnetic resonance (CMR), and biomarkers are becoming increasingly important in VHD. According to the European Heart Rhythm Association (EHRA) expert consensus statement [5] CMR plays a central role in risk stratification for MVP patients and should include measurements of left ventricle (LV) size and function, MR severity, leaflet length/thickness, MAD characterization, and curling, and LGE assessment.

The impact of these CMR features has been analyzed in only a few publications [8–18] and to the best of our knowledge a systematic revision is lacking. Therefore, the aim of our study is to perform a systematic review and meta-analysis of studies of MVP patients undergoing CMR to investigate the features that could discriminate between patients with co-VAs and without (N-co-VAs), thus providing comprehensive evidence on how to best leverage CMR as a diagnostic tool for arrhythmic risk stratification in MVP.

### Protocol and registration

This systematic review and meta-analysis was conducted according to the guidelines of the Preferred Reporting Items for Systematic Reviews and Meta-Analysis statement [19]. PROSPERO ID number: CRD42023374185.

### Eligibility criteria

Studies were considered eligible if they met all the following inclusion criteria:

1. Patients with MVP who underwent CMR with the assessment of at least one of the following: (1) LV size and function, (2) MR severity, (3) leaflet length/thickness/prolapse distance, (4) MAD, (5) curling, (6) LGE (papillary and/or myocardial), and (7) T1 mapping.
2. Arrhythmic profile reported.
3. The association of at least one CMR parameter with the arrhythmic profile was reported.

The primary endpoint was co-VAs. Co-VAs comprised non-sustained ventricular tachycardia (NSVT), sustained ventricular tachycardia (SVT), ventricular fibrillation (VF), and aborted SCD (aSCD). A sub-analysis was performed to further divide co-VAs into two groups based on the presence of NSVT vs SVT, VF, and aSCD, and a sensitivity analysis was performed to investigate the heterogeneity of the studies.

### Information sources and search strategy

The databases utilized for this study included Excerpta Medica dataBASE, Medical Literature Analysis and Retrieval System Online (PubMed/MEDLINE), and Cochrane Central Register of Controlled Trials (CENTRAL). The search was conducted until March 1, 2023. The string utilized is reported in the Supplementary Material. In addition to the electronic search, a manual search was conducted on the reference lists of selected papers to find any additional research that met the eligibility criteria.

### Selection and data collection process and data extraction

The inclusion criteria were used by two researchers, F.P. and A.S., in a two-stage approach to search for studies. Initially, they examined the title and abstract of the publications, followed by a review of the complete text. The rationales for the exclusion of studies during this subsequent phase were recorded. A comparison was made between the results of the two searches, and any inconsistencies that arose were then examined. In case of disagreement, the resolution was reached through the involvement of a third researcher (referred to as M.G.) in the process of consultation. The chosen articles were downloaded, imported, and de-duplicated in Microsoft Excel (Microsoft).

The following data was extracted: study title, authors, publication date, study design, number of patients enrolled, relevant baseline characteristics (including the definition of Co-VA with the respective standard of references used to evaluate it), CMR characteristics, and associations between CMR characteristics and study outcomes.

### Study risk of bias assessment

Two reviewers (F.P. and A.S.) separately assessed the quality of the selected studies, and any discrepancy was resolved by discussion and consensus. The risk of bias in each study—classified as low, moderate, or high—was scrutinized in terms of selection, performance, attrition, detection, reporting, and overall risk of bias, according to the guidelines of the Agency for Healthcare Research and Quality [20].

### Data analysis and synthesis

The association of each CMR parameter with co-VAs was meta-analyzed if reported in at least three studies. The association between the presence of CMR features and co-VAs was estimated by calculating Hedge’s g or pooled LogORs and their corresponding 95% CIs, when appropriate. Cochran’s *Q* test and *I*^2^ tests were used to measure heterogeneity between studies. For the *Q* test, *p* < 0.10 indicated significant heterogeneity; otherwise, heterogeneity was not statistically significant. For *I*^2^ tests, *I*^2^ between 0% and 25% was considered as low heterogeneity; *I*^2^ between 25% and 50% was considered as moderate heterogeneity, and *I*^2^ more than 50% was considered as high heterogeneity. Therefore, if *p* < 0.10 and *I*^2^ ≥ 50%, heterogeneity was present, and a random effect model was applied. Galbraith plots and Funnel plots were generated to assess heterogeneity within the included studies.

The sensitivity, specificity, positive (+ LR) and negative (− LR) likelihood ratios, and diagnostic odds ratio (DOR) with 95% confidence interval (CI) of dichotomous CMR parameters for the detection of co-VA were calculated using two-by-two contingency tables collected from each study. A bivariate random effects model was used to analyze, pool, and plot diagnostic performance measures from multiple studies. A hierarchical summary ROC curve (HSROC) was generated using logit values for sensitivity, specificity, and their respective variances. The clinical accuracy of each CMR parameter at the patient level was assessed using likelihood ratios to generate post-test probabilities based on Bayes’ theorem, Fagan’s nomograms, likelihood ratio scattergrams, and probability modifying plots. Stata (version 17.1, Stata Corp LP) was used to conduct the analyses.

## Results

**Fig. 1:**
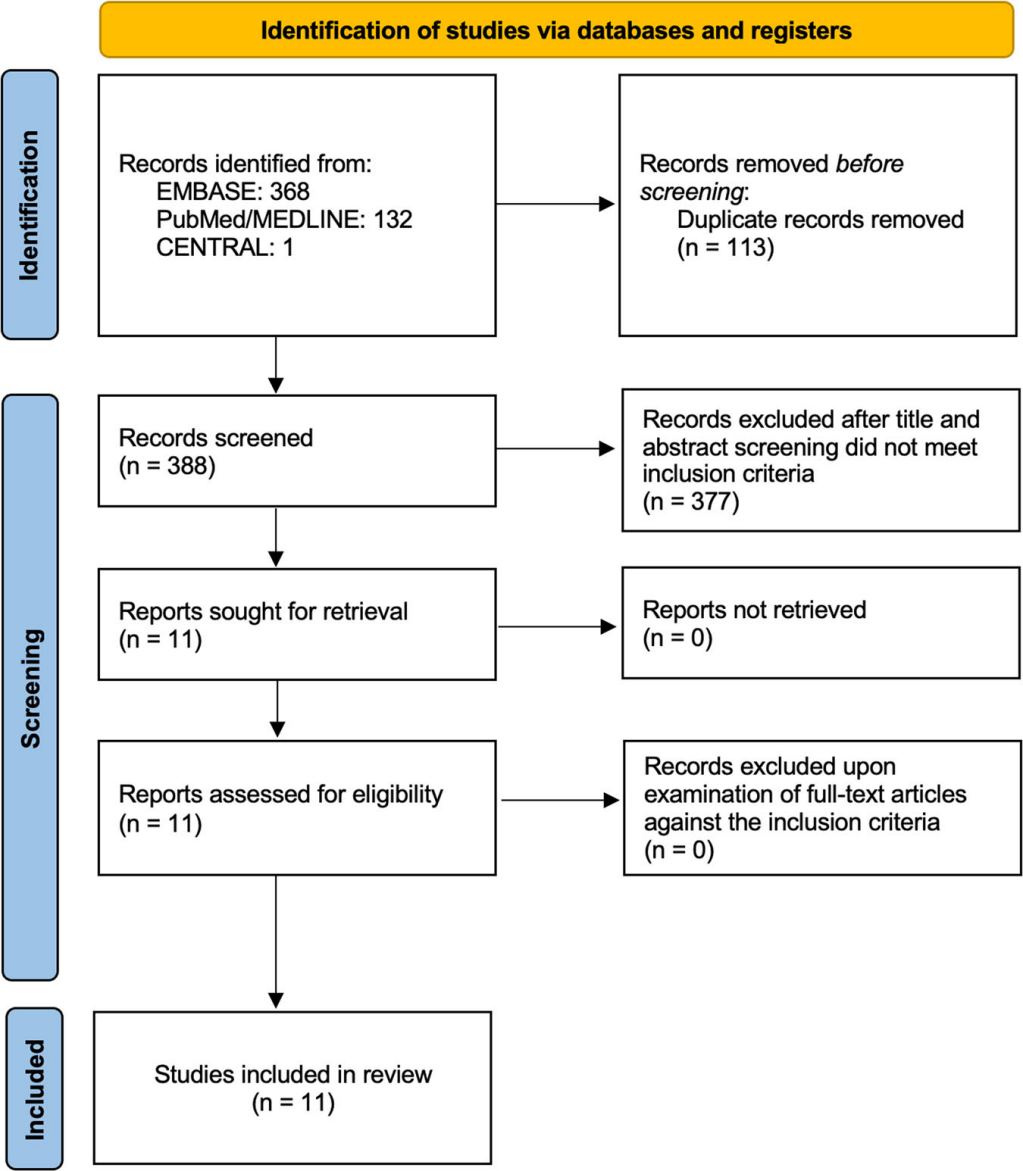
Consort diagram

A total of 388 papers were found in a search of electronic databases from their inception to March 1, 2023. Eleven studies with 1278 patients met the inclusion criteria. The consort diagram is shown in Fig. 1.

### Study characteristics and risk of bias

Table 1 summarizes the included studies, while Table 2 provides detailed baseline characteristics. The study period ran from 2015 to 2022, with one prospective and nine retrospective studies.

**Table 1:** Panel A: summary of the studies included. Panel B: summary of the studies included

Authors	Year	Study design	Inclusion criteria	Exclusion criteria	Control group
Basso et al	2015	Prospective	MVP patients were referred to the cardiology clinic from January 2010 to December 2013 with co-VAs.	Significant MR, tricuspid dysplasia or regurgitation, cardiomyopathies or congenital heart abnormalities, hemodynamically unstable conditions, and contraindications to CMR.	The control group consisted of patients with MVP with minor VA, that is, isolated VPB, couplets, and bigeminal VP.
Perazzolo Marra et al	2016	Not specified	Consecutive arrhythmic patients (either right bundle branch block or polymorphic VA) were referred to our cardiology clinic from January 2010 to July 2014 with echocardiographic diagnosis of MVP and underwent CE-CMR for the identification of LGE.	Moderate-to-severe MR, tricuspid dysplasia or regurgitation, cardiomyopathies or congenital heart abnormalities, hemodynamically unstable conditions, and contraindications to CE-CMR.	
Bui et al	2017	Retrospective	The electronic CMR database identified 44 patients with MVP referred for clinical CMR from 2006 to 2011.	Three studies with poor LGE CMR images were excluded. None of the patients with MVP had coronary artery disease, hypertension, or other intrinsic cardiomyopathies.	Consecutively enrolled healthy volunteers free of significant cardiac disease based on clinical and CMR findings.
Pradella et al	2018	Retrospective	All patients with MVP had undergone a complete CMR between December 2015 and December 2016.	MVP patients with other heart conditions (including other valvular problems).	
Enriquez A	2018	Retrospective	Patients undergoing catheter ablation of VAs between January 2012 and December 2015 in which one or more clinical PVC morphologies were mapped to the left ventricular (LV) PMs.	Acute ischemia, drug intoxication, electrolyte imbalance, mechanical MV prosthesis, and patients who had no spontaneous or inducible PVCs at the time of the electrophysiology study, precluding activation mapping, were excluded.	
Wang TKM et al	2021	Retrospective	21 Patients with MAD and MVP and 21 with MVP without MAD who underwent CMR and TTE within 6 months of each other were identified retrospectively.	Age < 18 years, history of cardiomyopathy, cardiac surgery, heart transplantation, congenital heart disease, and concurrent valve disease of at least moderate severity other than MR.	21 Controls without MVP or MAD who underwent CMR and TTE within 6 months of each other were identified retrospectively.
Constant Dit Beaufils et al	2021	Retrospective	Patients with MVP with trace to severe MR enrolled either in an MVP genetic study (genetic and phenotypic characteristics of MVP; NCT03884426) in Nantes (*n* = 293) or in the MVP STAMP study (stretch and myocardial characterization in arrhythmogenic MVP; NCT02879825) in Nancy, France (*n* = 152).	Previous cardiac surgery, an implanted device, nonischemic cardiomyopathy, congenital cardiac disease other than isolated bicuspid aortic valve, or more than mild aortic regurgitation or stenosis were not included in the study. Patients with a history of myocardial infarction or symptoms of coronary artery disease, with myocardial infarction-type fibrosis were excluded. Patients with coronary artery disease diagnosed by invasive coronary angiography during the preoperative workup were also excluded. Chronic kidney disease was not an exclusion criterion, but a contrast agent was not used during CMR if the creatinine clearance was < 30 mL/min/1.73 m^2^.	
Pavon et al	2021	Retrospective	From the CMR registry of the Lausanne University Hospital, we retrospectively included Patients with a bileaflet MVP and MAD between January 2011 and October 2019.	Patients with myocardial infarction, myocarditis, hypertrophic cardiomyopathy, infiltrative heart disease, more than mild associated VHD, and/or a LVEF < 50% were excluded.	As a control group, we included patients with various degrees of MR identified by echocardiography, who underwent CMR during the same period.
Gatti et al	2021	Retrospective	Consecutive patients with ages from 18 to 80 years and echocardiographic diagnosis of bMVP.	(i) Tricuspid, pulmonary, and aortic valve diseases; (ii) previous mitral valve surgery or percutaneous treatment; (iii) ischemic heart disease, cardiomyopathies, shunt, pulmonary hypertension, and aortic root dilatation; (iv) atrial fibrillation; (v) ICD or pacemaker; (vi) inability to hold breath or to lay down for 45 min; (vii) claustrophobia; (viii) recent history of alimentary/alcoholic/respiratory intoxication; (ix) estimated glomerular filtration rate < 30 mL min/1.73 m2; (x) history of allergic reaction to MR contrast media; and (xi) pregnancy or breastfeeding.	
Lee et al	2021	Retrospective	Patients who were diagnosed with MVP on echocardiography, a total of 117 patients (aged ≥ 18 years) patients who underwent CMR for any reason from January 2000 to June 2019 in three university hospitals were retrospectively included.	(1) Presence of concomitant structural heart disease other than MVP; (2) presence of possible causes of SCA other than MVP; (3) CMR performed after mitral valve surgery; and (4) significant (intervention-requiring) coronary artery disease.	
Figliozzi et al	2022	Retrospective	Aged 18 years or older, MVP was diagnosed at cardiac MRI, clinical information and continuous electrocardiogram monitoring were available within 3 months from cardiac MRI examination, and LGE imaging was available.	Cardiomyopathy, LV ejection fraction less than 40%, ischemic heart disease, congenital heart disease, inflammatory heart disease, moderate or worse MR (per transthoracic echocardiography report or MR fraction (20% at cardiac MRI), participation in competitive sport, or 12-lead electrocardiogram suggestive of channelopathies.	

Authors	Arrhythmia evaluation		CMR characteristics evaluated								
	Definition of Co-VA	Standard of reference	LV EDVi	LV EF	LA volume	MR severity	PD	MAD	Curling	LGE myocardial	LGE papillary
Basso et al	VF and ventricular tachycardia, either nonsustained or sustained	12-Lead ECG, 12-lead 24-h Holter monitoring	Yes	No	No	Yes	Yes	No	No	Yes	Yes
Perazzolo Marra et al	VF and ventricular tachycardia, either nonsustained or sustained	12-Lead ECG, 12-lead 24-h Holter monitoring	No	No	No	No	No	No	No	Yes	No
Bui et al	Grade III or higher by the Lown and Wolf classification	Holter or event monitor arrhythmia data	Yes	Yes	No	Yes	No	No	No	Yes	No
Pradella et al	Grade III or higher by the Lown and Wolf classification	12-Lead 48- to 72-h ECG Holter monitoring system (Mortara H12) was requested because of the presence of arrhythmic symptoms or 12-lead ECG changes.	No	No	No	No	No	No	No	Yes	No
Enriquez A	VF and ventricular tachycardia	Arrhythmia was evaluated during an electrophysiology study with detailed ECG analysis performed offline on the Prucka CardioLab recording system (GE, Houston, TX) with the recordings displayed at a speed of 100 mm/s.	No	No	No	No	No	No	No	No	Yes
Wang TKM et al	Ventricular tachycardia and/or VF	VA was defined as documented history in the electronic medical record of testing showing and/or hospitalization for ventricular tachycardia and/or VF.	No	No	No	No	No	Yes	No	No	No
Constant Dit Beaufils et al	NSVT and life-threatening ventricular arrhythmia	24-h ECG recording	No	No	No	No	No	No	No	Yes	No
Pavon et al	Grade 4 or 5 according to Lown classification	24 h Holter monitoring	No	No	No	No	No	No	No	Yes	No
Gatti et al	NSVT, SVT, or VF	12-Lead ECG, as well as 24-h Holter monitoring	Yes	Yes	Yes	Yes	No	Yes	Yes	Yes	Yes
Lee et al	VF and sustained or NSVT	12-Lead ECG	Yes	Yes	Yes	No	Yes	Yes	Yes	Yes	Yes
Figliozzi et al	VF and ventricular tachycardia, either nonsustained or sustained	At the study outset or with ambulatory electrocardiogram monitoring (24-hour monitoring) or implantable loop recorder within 3 months after cardiac MRI examination.	No	No	No	No	No	Yes	No	Yes	No

*CO-VA* complex ventricular arrhythmias, *LVEDVi* left ventricle end-diastolic volume index, *LVEF* left ventricle ejection fraction, *LA* left atrium, *MR* mitral regurgitation, *PD* prolapse distance, *MAD* mitral annular disjunction, *LGE* late gadolinium enhancement, *ECG* electrocardiogram, *NSVT* Non-sustained ventricular tachycardia, *VF* Ventricular fibrillation, *SVT* sustained ventricular tachycardia, *VA* ventricular arrhythmias

**Table 2:** Baseline characteristics

Author	Year	*N*		Age	Men	BMI	HT	DM	HC	Smoke
		Nr.	Prevalence of Co-VA	Years	%	%	%	%	%	%
Basso et al	2015	44	68.2%	46.8	35	NR	NR	NR	NR	NR
Perazzolo Marra et al	2016	52	69.2%	44	37	NR	NR	NR	NR	NR
Bui et al	2017	41	43.8%	52.8	61.7	NR	NR	NR	NR	sì
Pradella et al	2018	34	32.4%	56	59	NR	0	0	20	41
Enriquez A et al	2018	9	22.2%	54.7	36	NR	NR	NR	NR	NR
Wang TKM et al	2021	42	14.3%	54	57	24	28.5	2.3	21.4	NR
Constant Dit Beaufils et al	2021	400	15.8%	53	55	23.5	23	3	14	22
Pavon et al	2021	30	33.3%	50	60	NR	7	NR	7	10
Gatti et al	2021	52	38.5%	47.7	28.8	NR	11.5	0	9.6	9.6
Lee et al	2021	85	8.2%	54	54.1	22.7	25.9	8.2	9.4	NR
Figliozzi et al	2022	474	16.2%	46.8	48.5	NR	15.2	1.1	12.7	9.7

The studies included a total of 1278 patients (median per study 52 (interquartile range 38–69.5) patients). Four studies with 213 patients were included in the evaluation of the left ventricular end-diastolic volume index (LVEDVI) and left ventricular ejection fraction (LVEF). Prolapse distance was assessed in three studies involving 181 patients. MAD was assessed in four studies involving a total of 653 patients. The presence of LGE was investigated in eight studies with 1173 patients, while the percentage of LGE was evaluated in three studies with 181 patients. Of note, only three studies with 556 patients assessed the predictive value of LGE specifically for the occurrence of SVT, VF, and aSCD).

Table 3 shows the assessment of bias for each study. On overall risk assessment analysis, only one study [12] was found to be at high risk. Of note, that study reported only the association between arrhythmia and LGE of papillary muscles and was not included in any meta-analysis.

**Table 3:** Risk of bias analysis

Study name	Study year	Selection bias	Performance bias	Detection bias	Reporting bias	Overall bias
Basso et al	2015	Low	Low	Low	Low	Low
Perazzolo Marra et al	2016	Low	Low	Low	High	Low
Bui et al	2017	Low	Low	Low	Intermediate	Low
Pradella et al	2018	High	High	Low	Intermediate	Intermediate
Enriquez A et al	2018	High	High	High	High	High
Wang TKM et al	2021	High	Low	Intermediate	Low	Intermediate
Constant Dit Beaufils et al	2021	Low	Low	Low	Low	Low
Pavon et al	2021	Low	Low	Low	Low	Low
Gatti et al	2021	Low	Low	Low	Low	Low
Lee et al	2021	Low	Intermediate	Low	Low	Intermediate
Figliozzi et al	2022	Low	Low	Low	Low	Low

### Risk stratification

**Fig. 2:**
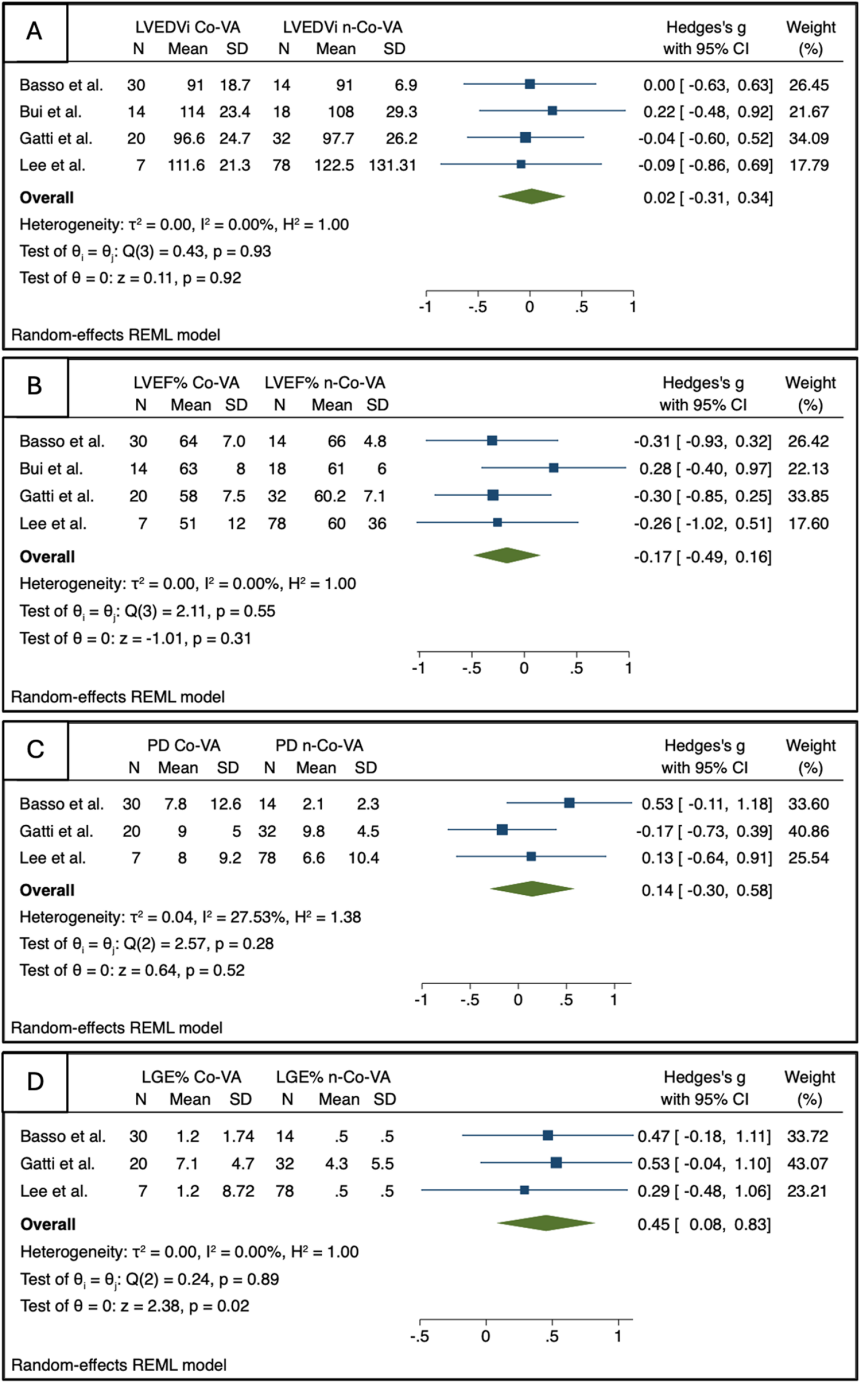
Summary forest plots for the association between CMR characteristics (LVEDVi (**A**), LVEF% (**B**), PD (**C**), and LGE% (**D**), and the presence of complex ventricular arrhythmia

**Fig. 3:**
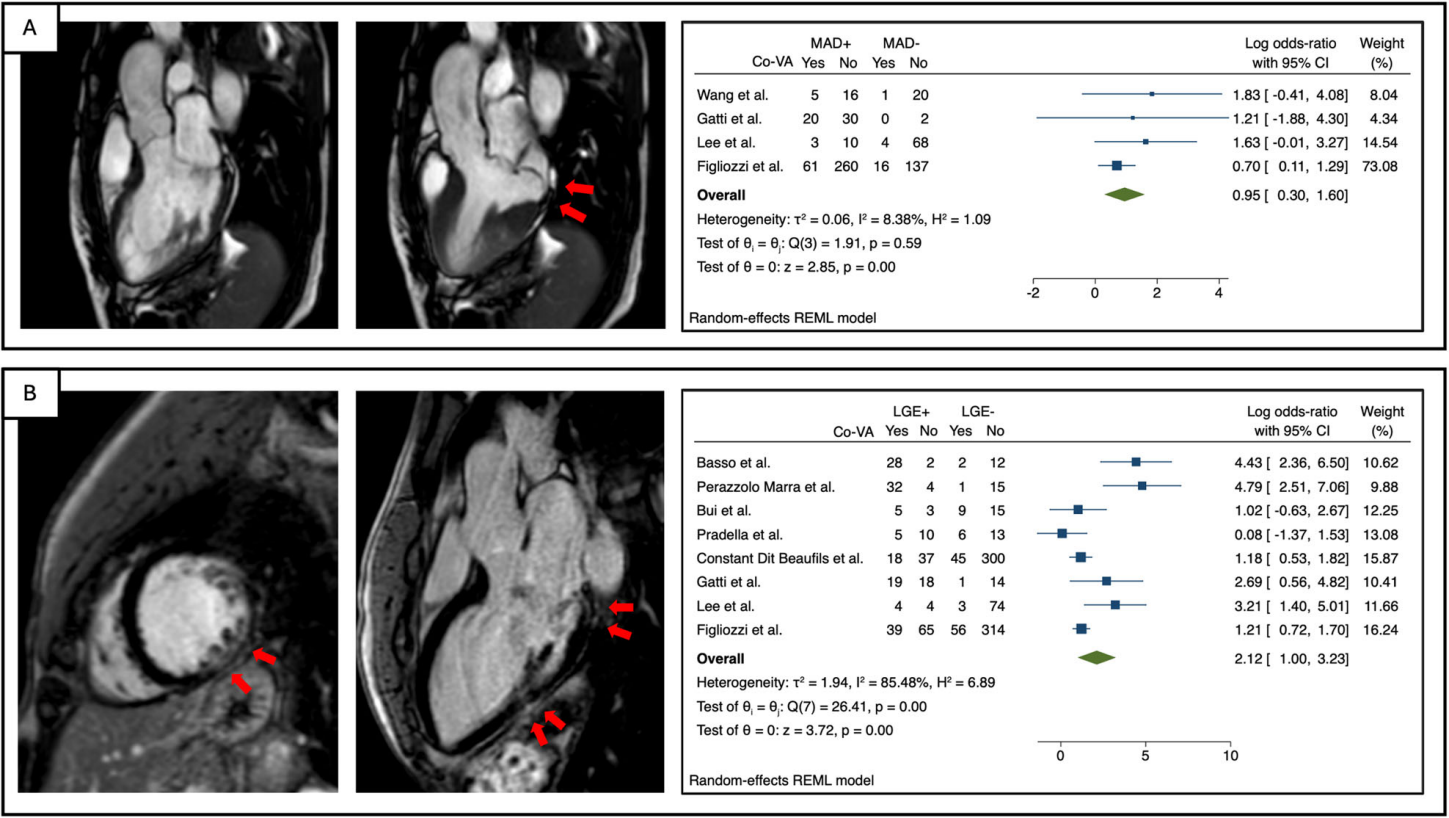
CMR images and summary forest plots for the association between CMR characteristics and the presence of complex ventricular arrhythmia (**A** MAD and **B** LGE)

The association of CMR parameters with Co-VAs was largely different in terms of effect size and CI for different CMR parameters. MR severity, leaflet length/thickness, curling, MAD distance, and mapping techniques were not included in the meta-analysis, as they were reported in fewer than three studies. The Hedge’s g values for LVEDVi, LVEF, and prolapse distance were 0.02 (95% CI: [−0.31, 0.34]), −0.17 (95% CI: [−0.49, 0.16]), and 0.14 (95% CI: [−0.30, 0.58]), respectively, indicating small effect sizes with no statistical significance (Fig. 2A–C)). For MAD, the effect size was significant with a log odds ratio (OR) of 0.95 (95% CI: [0.30, 1.60]), while for LGE, the log OR was 2.12 (95% CI: [1.00, 3.23]) (Fig. 3A, B). The percentage of LGE showed a Hedge’s g of 0.45 (95% CI: [0.08, 0.83]) (Fig. 2D)

**Fig. 4:**
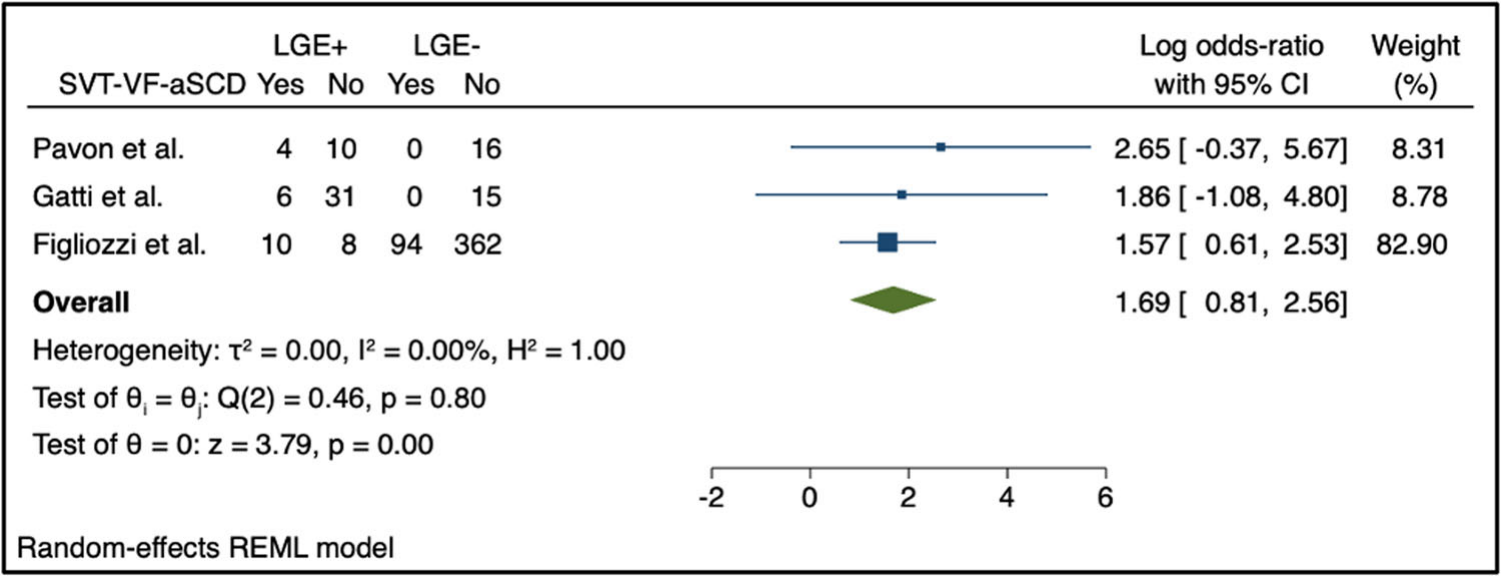
Summary forest plots for the association between CMR characteristics (LGE) and the presence of SVT, VF, or aSCD

Sub-analysis to further stratify the arrhythmic risk of patients into two groups based on the presence of SVT, VF, and aSCD was only possible for LGE, which showed a log odds-ratio of 1.69 (95% CI: [0.81, 2.56]) (Fig. 4).

Details of the sensitivity analysis conducted to assess study heterogeneity are provided in the Supplementary Materials.

### Sensitivity, specificity, and diagnostic performance

**Fig. 5:**
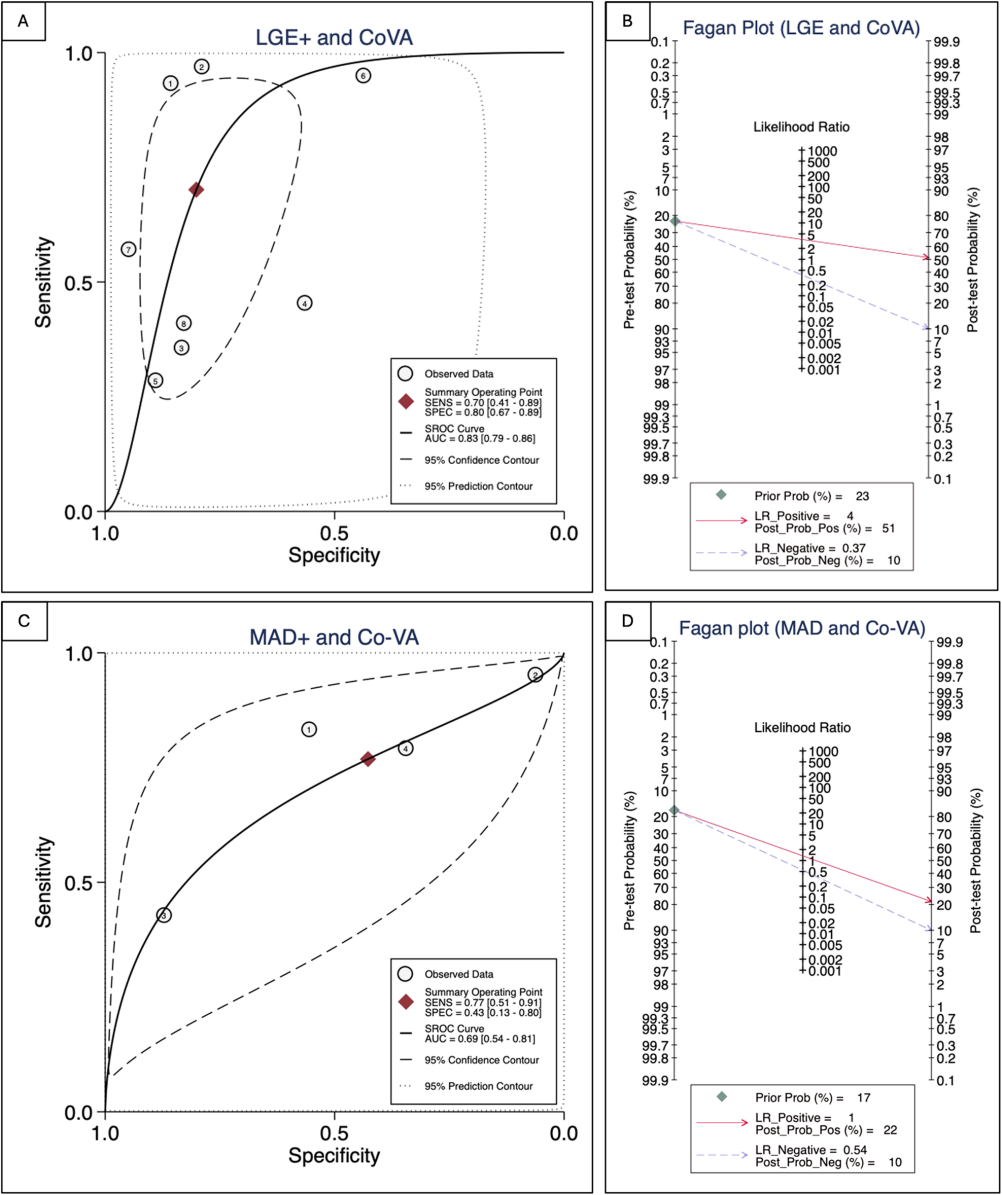
Hierarchical summary receiver operating characteristic curve plot (**A**, **C**) and Fagan Plot (**B**, **D**) for LGE and MAD

For LGE, the HSROC AUC was 0.83 (CI: 0.69, 0.91), indicating good overall predictive accuracy (Fig. 5A). Sensitivity and specificity were 0.70 (CI: 0.41, 0.89) and 0.80 (CI: 0.67, 0.89), respectively. The positive likelihood ratio for LGE was 3.5 (CI: 2.1, 5.9) indicating a small increase in the likelihood of disease after test discrimination. Conversely, the negative likelihood ratio was 0.37 (CI: 0.17, 0.83) suggesting a small decrease in the likelihood of disease after test discrimination. The DOR was 9 (CI: 3, 29), demonstrating the discriminatory ability of LGE. Fagan’s nomogram, with a pre-test probability for Co-VAs of 23%, showed a post-test probability of 51% in the presence of LGE and 10% in the absence of LGE (Fig. 5B).

For MAD, the HSROC AUC was 0.69 (CI: 0.54, 0.81), with a sensitivity of 0.77 (CI: 0.51, 0.91) and a specificity of 0.43 (CI: 0.13, 0.80) (Fig. 5C). The positive and negative likelihood ratios were 1.3 (CI: 0.8, 2.2) and 0.54 (CI: 0.36, 0.82), respectively. The DOR for MAD was 2 (CI: 1, 5), indicating lower overall diagnostic efficacy compared to LGE. According to Fagan’s nomogram for MAD, with a pre-test probability of 17%, the post-test probability was 22% if MAD was present and 10% if absent (Fig. 5D).

## Discussion

Our systematic review and meta-analysis evaluated the role of CMR in risk stratification of arrhythmia in patients with MVP.

The findings of the study emphasize the significance of LGE in the process of arrhythmic risk classification for patients diagnosed with MVP. Specifically, the presence of LGE demonstrated good overall predictive accuracy with balanced performance in identifying MVP patients at risk for Co-VA, with a Log OR of 2.12, positive likelihood ratio of 3.5, negative likelihood ratio of 0.37, sensitivity of 0.70, and specificity of 0.80 with an AUC of 0.83. Furthermore, LGE was also associated with SVT-VF-aSCD (log OR of 1.69), highlighting its utility in identifying MVP patients at higher risk of major arrhythmic events.

This observation is consistent with the growing body of evidence highlighting the predictive value of LGE for Co-VAs and SCD in MVP patients [8–10, 14, 16, 17, 21]. Indeed, higher extension of low voltages has been found in electrophysiological studies in the case of LGE involving papillary muscle and lateral/infero-lateral LV wall (in patients with MVP and complex arrhythmia, supporting the role of fibrosis as electrophysiological substrate [22].

Myocardial fibrosis in MVP seems to be the result of increased traction of the papillary muscles and mechanical stretch of the surrounding myocardium. LGE thus mainly involves the inferior and lateral basal wall of the LV, with a non-ischemic appearance (mid-wall or patchy) and less frequently with subendocardial pattern and the posteromedial papillary muscle [23]. Moreover, systolic curling of the mid-basal lateral wall may constitute a potential electromechanical trigger further enhancing the risk of VA [23].

Ever-increasing evidence supports the value of fibrosis assessment by LGE for arrhythmic risk stratification across several clinical settings. A recent meta-analysis conducted by Al-Sadawi et al [24] on more than ten thousand non-ischemic cardiomyopathy patients confirmed a significant 4.6 risk of VA and SCD among LGE-positive patients. In general, when evaluating a patient with MVP, it is essential to always consider other possible etiologies that may cause a similar pattern of LGE (e.g., myocarditis and coronary artery disease). Overall, our findings confirm and reinforce the value of LGE assessment for arrhythmic risk stratification also among patients with MVP.

Importantly, despite strong association with Co-VAs, a non-negligible proportion of events occurred also among patients without LGE, suggesting the multifactorial etiology of arrhythmias and supporting the need for further research to achieve a multiparametric risk stratification. Of note, interstitial fibrosis detected only by mapping techniques may partly explain this discrepancy. Indeed, increased native T1 and ECV values on the lateral wall in patients with MVP were associated with arrhythmic events even in the absence of LGE [15, 25]. However, we could not meta-analyze T1 mapping due to the limited study number available. Future studies will elucidate the addictive performance of these promising markers over LGE among MVP patients.

The analysis also supported the role of MAD in predicting arrhythmic risk. Although the presence of MAD showed a moderate association with co-VAs (log OR 0.95), with relatively high sensitivity (0.77) and negative likelihood ratio (1.3), the predictive value of MAD appears to be lower than that of LGE. Its low specificity (0.43) may indeed be related to the dichotomic evaluation of MAD rather than an evaluation based on MAD extension and site. In fact, a degree of MAD seems to be prevalent in the general population, possibly representing a normal variant of mitral annulus anatomy [26]. Conversely, MAD extension over a certain length, especially in the setting of MVP, resulted associated with Co-VAs and SCD, with pathological MAD reported for length higher than 5 mm or 8.5 mm [9, 27], albeit a precise cut-off that remains to be determined, and when localized in the infero-lateral wall and associated with MVP [9, 23, 26]. Associated with MVP, longer MAD seems to worsen mitral annulus dynamics with subsequent more severe mechanical wall stretch and fibrosis, concurring in eliciting arrhythmias.

A multimodality approach to MVP patients is increasingly recognized in importance, especially for arrhythmic risk stratifications. Indeed, the value of continuous rhythm monitoring either with Holter-ECG or implantable loop recorders demonstrated the importance of VA burden and complexity in predicting life-threatening events [28]. These tools could be used comprehensively together with CMR, although their combined performance remains to be evaluated. Moreover, new CMR parameters may complement LGE evaluation.

Contrary to previous studies [8, 10], our analysis showed that the prolapse distance, LVEDVi, and LVEF were not significantly associated with co-VAs. This discrepancy could be due to differences in patient selection criteria across studies. However, it also underscores the complexity of MVP and the multifactorial nature of its associated arrhythmic risks. Therefore, a single parameter might not be sufficient to capture the nuanced risk profile of an MVP patient.

Overall, our findings confirm the usefulness of a CMR approach in arrhythmic risk stratification in MVP patients, as recommended by the ESC/EACTS guidelines by the EHRA expert consensus statement [5, 7]. Given the multifaceted nature of MVP-related arrhythmias, combining several CMR features, particularly LGE and MAD features, might provide a more comprehensive and accurate risk assessment.

Some limitations of this analysis must be acknowledged. Due to the retrospective nature of the majority of included studies, inherent bias cannot be ruled out. In addition, heterogeneity in study design and patient populations may have influenced the results, however, only one study was assessed as having a high risk of bias. Moreover, we could not analyze the impact of MR severity, leaflet length/thickness, curling, MAD distance, and mapping techniques because a limited number of studies were available. This underscores the importance of further research to assess whether these parameters may improve current well-validated risk stratification markers. Finally, no attempt was made to find unpublished or grey data, as the focus of this study was solely on published literature.

In conclusion, this systematic review and meta-analysis emphasize the value of LGE as a key CMR characteristic for assessing arrhythmia risk classification in patients with MVP. Moreover, the presence of MAD may further stratify these patients. Thus, a multi-parametric CMR approach provides a more comprehensive risk assessment. Future prospective multicenter studies are needed to validate these findings and to establish standardized guidelines for the integration of CMR in the management of MVP. Finally, while CMR is a useful tool for risk stratification, its results must always be linked with clinical parameters and individual patient features for a thorough risk assessment.

## Supplementary information

Electronic Supplementary Material

## Abbreviations

AMVP: Arrhythmic mitral valve prolapse
aSCD: Aborted sudden cardiac death
AUC: Area under the curve
CENTRAL: Cochrane Central Register of Controlled Trials
CI: Confidence interval
CMR: Cardiac magnetic resonance
co-VAs: Complex ventricular arrhythmias
DOR: Diagnostic odds ratio
EACTS: European association for cardio-thoracic surgery
ECG: Electrocardiogram
EHRA: European Heart Rhythm Association
ESC: European Society of Cardiology
HSROC: Hierarchical summary receiver operating characteristic
LGE: Late gadolinium enhancement
LR: Positive likelihood ratio
−LR: Negative likelihood ratio
LV: Left ventricle
LVEDVI: Left ventricular end diastolic volume index
LVEF: Left ventricular ejection fraction
MAD: Mitral annular disjunction
MR: Mitral regurgitation
MVP: Mitral valve prolapse
N-co-VAs: Non-complex ventricular arrhythmias
NSVT: Non-sustained ventricular tachycardia
OR: Odds ratio
PD: Prolapse distance
SCD: Sudden cardiac death
SVT: Sustained ventricular tachycardia
VF: Ventricular fibrillation
VHD: Valvular heart disease
