# Supplementary material for: Role of cardiac magnetic resonance in stratifying arrhythmogenic risk in mitral valve prolapse patients: a systematic review and meta-analysis
Source: Eur Radiol. 2024 Jun 6;34(11):7321–33. doi: 10.1007/s00330-024-10815-3 (PMC11519141; doi:10.1007/s00330-024-10815-3)

Role of cardiac magnetic resonance in stratifying arrhythmogenic risk in mitral valve prolapse patients: a systematic review and meta-analysis

Electronic Supplementary Material (ESM)

Eur Radiol (2024) Gatti M, Santonocito A, Papa FP et al.

Search strategy – string

PubMed/MEDLINE

(“mitral valve prolapse” [mh] OR mitral valve prolapse [tw]) AND (“Magnetic Resonance Imaging" [mh] OR Magnetic Resonance Imaging [tw] OR mri [tw] OR MR Imag* [tw] OR Cardiac Magnetic Resonance [tw] OR CMR [tw] OR NMR imag* [tw] OR MR tomograph* [tw]) NOT ("case reports"[Publication Type] OR "comment"[Publication Type] OR "editorial"[Publication Type] OR "letter"[Publication Type] OR animal)

Excerpta Medica dataBASE (EMBASE),

('mitral valve prolapse'/exp OR 'mitral valve prolapse') AND ('cardiovascular magnetic resonance'/exp OR 'cardiovascular magnetic resonance') AND ('heart arrhythmia'/exp OR 'heart arrhythmia')

Cochrane Central Register of Controlled Trials (CENTRAL)

('mitral valve prolapse') AND ('cardiovascular magnetic resonance') AND ('heart arrhythmia')

Sensitivity — heterogeneity analysis

Investigating the heterogeneity of the studies, the only analysis with high heterogeneity was the analysis of LGE for predicting the presence of Co-VAs with τ^2=1.94 I^2=85.48 and H^2 equal to 6.89. Therefore, both the Galbraith plot (figure s1), to further evaluate heterogeneity, and the Funnel plot, to evaluate publication bias (figure s2), were constructed, identifying two studies that fell outside the "normal range". The analyses were then re-performed excluding these studies, finding for the association of LGE with Co-VAs a log odds ratio of 1.24 (95% CI [0.89, 1.60]) with τ^2=0.00, I^2=0.00 and H^2 equal to 0.00 (supplementary figure s3).

Additionally sensitivity, specificity, and diagnostic performance of LGE were reassessed excluding the aforementioned outlier studies. The sensitivity and specificity were determined to be 0.48 (CI: 0.28, 0.69) and 0.80 (CI: 0.64, 0.90), respectively. The positive likelihood ratio for LGE was 2.4 (CI: 1.7, 3.1), indicating a small increase in the likelihood of disease after test discrimination. Conversely, the negative likelihood ratio was 0.65 (CI: 0.49, 0.86), suggesting only a minimal change in the likelihood of the disease after test discrimination. The diagnostic odds ratio was found to be 3.6 (CI: 2.5, 5.3), demonstrating the discriminatory ability of LGE.

FIGURE LEGENDS

Figure s1. Galbraith plot for the association between LGE and the presence of complex ventricular arrhythmia.

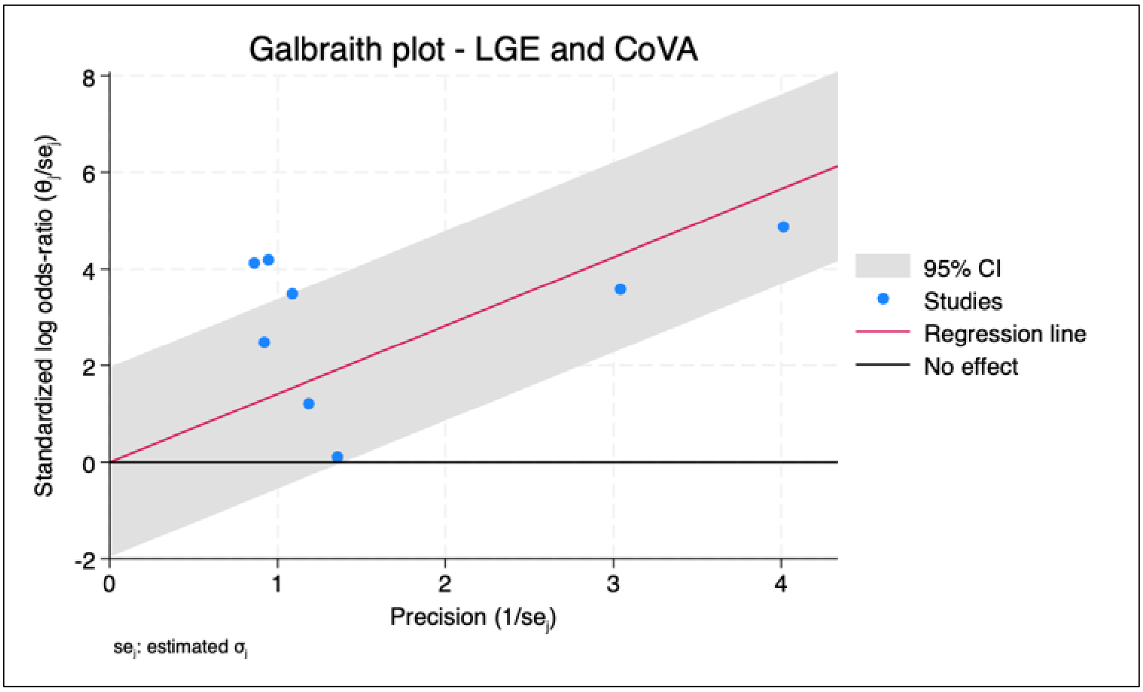

Figure s2. Funnel plot for the association between LGE and the presence of complex ventricular arrhythmia.

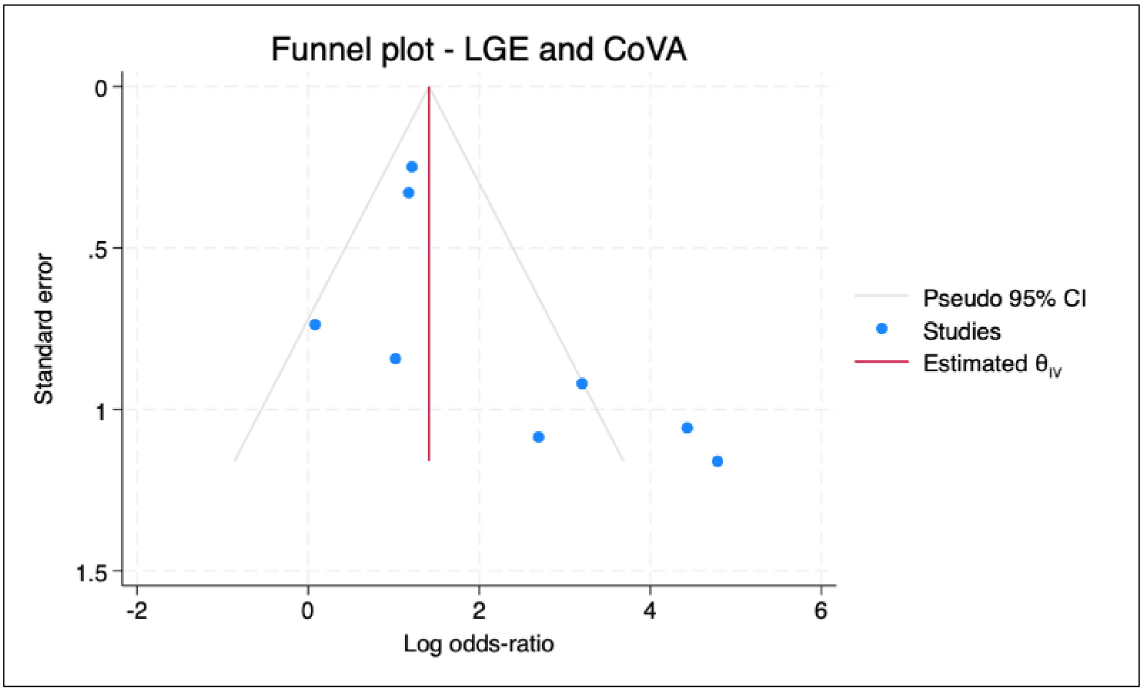

Figure s3. Summary forest plots for the association between LGE and the presence of complex ventricular arrhythmia excluding studies with high heterogeneity.

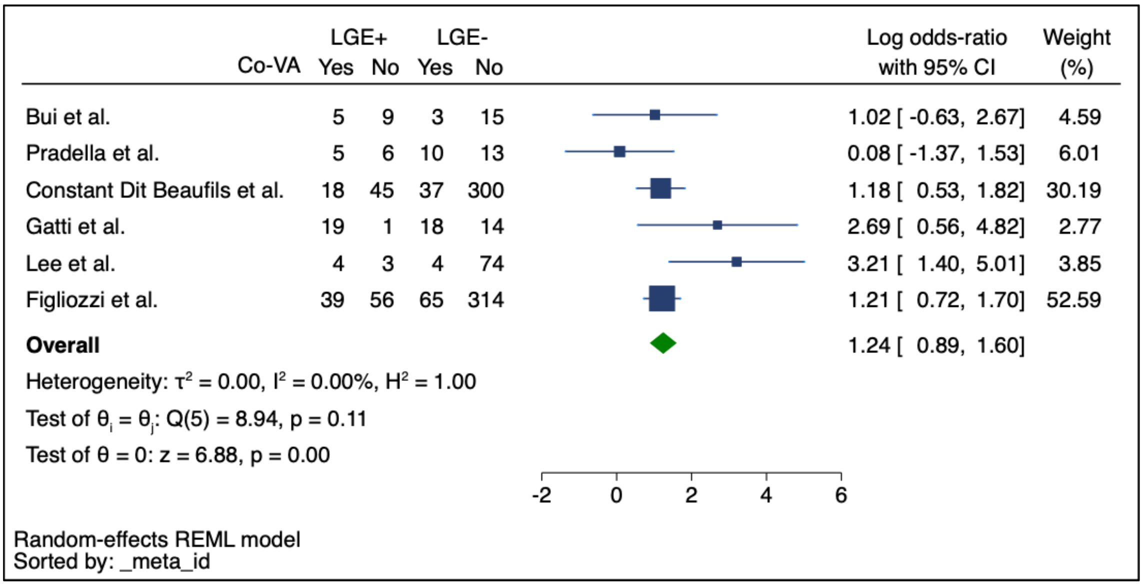

Supplement: Supplementary file 1 — Electronic Supplementary Material [file 330_2024_10815_MOESM1_ESM.pdf]
